# LncRNA-H19 regulates chemoresistance to carboplatin in epithelial ovarian cancer through microRNA-29b-3p and STAT3

**DOI:** 10.7150/jca.58979

**Published:** 2021-07-25

**Authors:** Xueye Tian, Xiaohang Zuo, Meng Hou, Chen Li, Yue Teng

**Affiliations:** 1 Department of Obstetrics and Gynaecology, The First Affiliated Hospital of Xi'an Jiaotong University, Xi'an 710061, China; 2 Xijing Hospital, Fourth Military Medical University, Xi'an 710032, China

**Keywords:** lncRNA-H19, miR-29b-3p, STAT3, carboplatin resistance, epithelial ovarian cancer

## Abstract

**Background:** Platinum-based chemotherapy is part of current standard treatment for epithelial ovarian cancer (EOC). However, chemoresistance often rapidly developed, leading to chemotherapy failure and unfavored prognosis. Increasing evidence has demonstrated the important role of oncogenic long noncoding RNA H19 in various cancers, including EOC. No current study is available in exploring the role of lncRNA-H19 in carboplatin resistance of EOC and its underlying mechanism.

**Methods:** Levels of lncRNA-H19, miR-29b-3p, and STAT3 mRNA were measured by qRT-PCR. The 50% inhibitory concentration value was detected with Cell Counting Kit-8 (CCK8). Colony-formation and CCK8 assays were employed to measure cell viability. Cell migration and invasion ability was evaluated with transwells. Western blot assay was utilized to measure P-gp, MRP1, LRP, and STAT3 protein levels. The targeting between lncRNA-H19 and miR-29b-3p, as well as miR-29b-3p and STAT3, was verified by dual-luciferase, RNA immunoprecipitation, and RNA pull-down experiments.

**Results:** lncRNA-H19 and STAT3 were sharply increased, while miR-29b-3p was decreased in carboplatin-resistant EOC. Carboplatin efficacy was enhanced by lncRNA-H19 silencing in chemo-resistant EOC cells. lncRNA-H19 served as a competing endogenous RNA of miR-29b-3p, causing the derepression of miR-29b-3p downstream target STAT3, leading to chemoresistance in carboplatin-tolerated EOC.

**Conclusions:** The lncRNA-H19/miR-29b-3p axis improved carboplatin resistance of EOC by targeting STAT3, indicating a possible approach to improving chemotherapy for EOC.

## Introduction

Epithelial ovarian cancer (EOC) is the most malignant and lethal gynecological disease and is characterized by the early onset of disseminated metastasis. Cytoreduction and platinum-based chemotherapy constitute the current standard treatment for EOC, which is effective when treatment begins. However, as treatment continues, most EOC patients become resistant to chemotherapy 1. Exploration of the underlying molecular mechanism of chemoresistance is urgently needed for effective treatment for EOC.

Noncoding RNAs refer to a large class of RNA transcripts with no ability to encode proteins that have recently emerged as promising regulatory molecules in various biological and pathological processes, including X-chromosome deactivation, stem cell revival, cellular differentiation, apoptosis and invasion 2 3 4. According to their lengths, regulatory ncRNAs are categorized into small ncRNAs (<200 nt) and long noncoding RNAs (lncRNAs; ≥200 nt) 5. Intriguingly, different types of ncRNAs are found to interact with each other to exert their functions. For instance, lncRNAs have been found to regulate microRNAs (miRNAs) by serving as competing endogenous RNAs, and miRNAs are often found to regulate gene expression by binding to mRNAs at their 3' untranslated regions (UTRs) 6. Among a variety of identified lncRNAs, lncRNA-H19 has been found to be differentially expressed and participate in the onset and development of ovarian cancer 7 8. Some studies have shown that the level of lncRNA-H19 is abnormally elevated in ovarian cancer tissues, and H19 may be involved in the cisplatin resistance of EOC cells 9. To date, there is no study available on the role of lncRNA-H19 in EOC carboplatin resistance. In our earlier studies, we explored the biological functions of miR-29b-3p in EOC development and found that miR-29b-3p is an important regulator of energy metabolism, EMT, and epigenetic mechanisms of EOC 10 11 12. miR-29b-3p was also identified as a target of lncRNA-H19 in recent studies 9. Considering the involvement of lncRNA-H19 and miR-29b-3p in EOC progression, we aimed to determine whether the interaction between lncRNA-H19 and miR-29b-3p contributes to the regulation of chemoresistance in EOC. Notably, using bioinformatics software, we previously identified signal transducer and activator of transcription 3 (STAT3) as a predicted miR-29b target, suggesting that miR-29b-3p participates in regulation of chemoresistance via the STAT3 pathway. Currently, no research has explored the role of the lncRNA-H19-miR-29b-3p-STAT3 axis in EOC chemoresistance. Elucidating the role of the lncRNA-H19-miR-29b-3p-STAT3 axis in EOC chemoresistance will help to better understand EOC and provide novel clinical therapies for EOC.

## Materials and Methods

### Cell lines and human tissue specimens

The human ovarian cancer cell line SKOV3 (obtained from ATCC, Manassas, VA, USA) and the carboplatin-resistant human ovarian cancer cell lines SKOV3-CB (obtained from Cancer Research Institute of Guangxi Medical University) were maintained in high-glucose DMEM (Gibco, Invitrogen, Carlsbad, CA, USA) supplemented with 10% (v/v) fetal bovine serum (Gibco, Invitrogen, Carlsbad, CA, USA) at 37°C in a humidified 5% CO_2_ atmosphere 13. SKOV3-CB cells were preserved in a final concentration of 5 nmol/L carboplatin to maintain their resistance. This study was approved by the Ethics Committee of The First Affiliated Hospital of Xi'an Jiaotong University, China (study reference No.: XJTU1AF2018LSK-245). Written consent was obtained from each study participant enrolled. twenty high-grade serous ovarian cancer samples and twenty normal ovarian tissue samples were used in our study. These samples were all newly diagnosed, previously untreated, and collected from patients at The First Affiliated Hospital of Xi'an Jiaotong University, PR China between 2013 and 2014. Detailed diagnostic and pathological reports were collected for all patients, and none of them had been previously treated with chemotherapy.

### Bioinformatics analysis

Potential targets and binding sites of miR-29b-3p were predicted using several online programs, including miRanda (http://www.microrna.org/microrna/home.do) and TargetScan (http://www.targetscan.org/); STAT3 was predicted to be a downstream target gene of miR-29b-3p by both software programs, and this finding was further validated by experiments.

### Plasmids and cell transfection

Short interfering RNA (siRNA) for lncRNA-H19 (si-H19; 50 nM), negative control siRNA (si-NC; 50 nM), lncRNA-H19 overexpression plasmid (pcDNA3.1‑H19; 2 µg), pcDNA3.1 (2 µg), miR‑29b‑3p mimic (60 nM), miR‑29b‑3p inhibitor (120 nM), STAT3-overexpression plasmid (pcDNA3.1-STAT3; 2 µg), STAT3 siRNA (si‑STAT3, 2 µg) and negative control (NC) were all purchased from RiboBio (Guangzhou, China). Ovarian cancer cells were seeded into 6-well plates until 50%-60% confluency and transiently transfected with targeted siRNAs using X-treme GENE siRNA Transfection Reagent (Roche, Indianapolis, IN, USA) according to the manufacturer's instructions. Forty-eight hours after transfection, the cells were collected for further experiments.

### Quantitative real-time PCR

PCR experiments were performed as indicated in our former studies 10. The primers for lncRNA-H19, miR-29b-3p and u6 reverse transcription and amplification were designed by and purchased from RiboBio Co., Ltd. (Guangzhou, China). The primer sequences were as follows: STAT3: F: GTCAGGCACCATTTTAGGCAC, R: TCAGGCTTTCCTTGACCAGT; lncRNA-H19: F: TACAACCACTGCACTACCTG, R: TGGAATGCTTGAAGGCTGCT; miR-29b-3p: F: ACACTCCAGCTGGGTAGCACCATTTGAAATC, R: TGGTGTCGTGGAGTCG; u6: F: CTCGCTTCGGCAGCACA; R: TGGTGTCGTGGAGTCG.

### Viability assay and IC50 determination

A total of 5 × 10^3^ cells were seeded in 96-well plates and treated with increasing concentrations of carboplatin (from 1000 µM to 15.625 µM by 2-fold dilution steps) or a combination of carboplatin (from 1000 µM to 15.625 µM by 2-fold dilution steps) and H19 (50 µM) 24 h after seeding. At 48 h or 72 h after treatment, cell viability was determined by a colorimetric assay using a tetrazolium salt, WST-1 (Roche). A nonlinear regression was performed, and the relative IC50 values were determined with GraphPad Prism.

### Colony-forming assay

Transfected SKOV3-CB cells were seeded into 6-well plates at a density of 500 cells per well. Following incubation at 37°C and 5% CO_2_ for 2 weeks, visible colonies were fixed and stained with 0.1% crystal violet (Sigma-Aldrich Co.), and the number of colonies was counted under an inverted microscope (Olympus, Tokyo, Japan).

### Cell migration and invasion assay

In vitro cell migration assays were performed using Transwell chambers (8 μM pore size; Costar). After reaching 75%-80% confluency, cells were serum-starved overnight. After being detached with trypsin and washed with PBS, the cells were then resuspended in serum-free medium, and 500 μl cell suspensions (5 × 10^5^ cells/ml) were added to the upper chamber. Complete medium was added to the bottom chambers. The cells that had not migrated were removed from the upper face of the filters using cotton swabs, while the migrated cells were fixed with 5% glutaraldehyde solution and stained with 0.5% solution of toluidine blue in 2% sodium carbonate. Images of five random ×10 fields were captured from each membrane, and the number of migratory cells was counted, with the mean value of five duplicate assays for each experimental condition used for statistical analysis. Similar inserts coated with Matrigel were used to determine invasive potential in the invasion assay.

### Western blot analysis

WB experiments were performed as indicated in our former studies 10. Mouse anti-human STAT3 (ab119352, Abcam, Cambridge, UK), rabbit anti-human P-gp (ab235954, Abcam, Cambridge, UK), mouse anti-human MRP1 (ab24102, Abcam, Cambridge, UK), rabbit anti-human LRP (ab92544, Abcam, Cambridge, UK), and mouse anti-human β-actin (#3700S, CST, MA, USA) were incubated with the membranes overnight at 4°C at dilutions of 1:800, 1:800, 1:800, 1:800, and 1:2000, respectively.

### Luciferase reporter assay

The putative binding regions of lncRNA-H19 and miR-29b-3p or miR-29b-3p and STAT3 were predicted by bioinformatics analysis 14. Briefly, it is predicted by starBase software that lncRNA-H19 could target miR-29b-3p. miRanda and TargetScan software predicted STAT3 as a downstream target of miR-29b-3p. Luciferase reporter assay was employed to confirm the binding of the genes. Partial sequences of lncRNA-H19 containing wild-type or mutant binding sites of miR-29b-3p were cloned into the psiCHECK™-2 plasmid (Promega Corporation, Fitchberg, WI, USA) to generate the wild-type plasmid (H19-WT) or mutant-type plasmid (H19-MUT). Similarly, wild-type or mutant plasmid STAT3 3ʹUTR-WT or STAT3 3ʹUTR-MUT was also constructed using the same approach. The luciferase reporter plasmid was transfected into SKOV3-CB cells together with miR-NC or miR-29b-3p. Relative firefly luciferase activity, normalized with Renilla luciferase, was then measured 48 h after transfection with a dual-luciferase reporter gene assay system (Promega, Madison, WI, USA), and the results are shown as the percentage change over the appropriate control. Since complementary binding between lncRNAs, miRNAs and mRNAs leads to decreased expression of the downstream gene, a decreased luciferase activity indicates the binding of specific gene sequences inside reporter plasmids.

### RNA immunoprecipitation (RIP)

RIP analysis was conducted in SKOV3-CB cells using the Magna RIP RNA-Binding Protein Immunoprecipitation Kit (EMD Millipore) according to the manufacturer's instructions. In brief, SKOV3-CB cells were transfected with miR-29b-3p or miR-NC for 48 h. Then, the cells were lysed in RIP buffer, and cell extraction was incubated with Protein A/G magnetic beads bound with primary antibodies against Ago2 (Abcam) or IgG (Abcam). The protein and DNA in the immunoprecipitated complex were removed, and the enrichment levels of lncRNA-H19 were measured by qRT-PCR.

### RNA pull-down

The interaction between lncRNA-H19 and miR-29b-3p was examined using the Pierce Magnetic RNA-Protein Pull-Down Kit (Thermo Fisher Scientific) according to the manufacturer's protocols. Biotin-labeled wild-type lncRNA-H19 (Bio-H19 WT) containing the putative miR-29b-3p binding sites and the biotin-labeled mutant lncRNA-H19 (Bio-H19 MUT) designed to disrupt the base pairing between lncRNA-H19 and miR-29b-3p were mixed with protein extracts of SKOV3-CB cells and M-280 streptavidin-coated magnetic beads (Invitrogen). The beads were subsequently washed with ice-cold lysis buffer, low-salt buffer, and high-salt buffer. Next, bound RNAs were purified from the RNA-protein complex, and miR-29b-3p expression was determined by qRT-PCR.

### Statistical analysis

Each experiment was independently performed at least 3 times. Data are presented as the mean ± standard deviation (SD) and were analyzed using GraphPad Prism software. Statistical significance was assessed using two-tailed unpaired Student's t-test. When a *P* value was less than 0.05, the differences were considered statistically significant.

## Results

### lncRNA-H19 expression characteristics in EOC and its correlation with the prognosis of EOC patients

**Figure 1:**
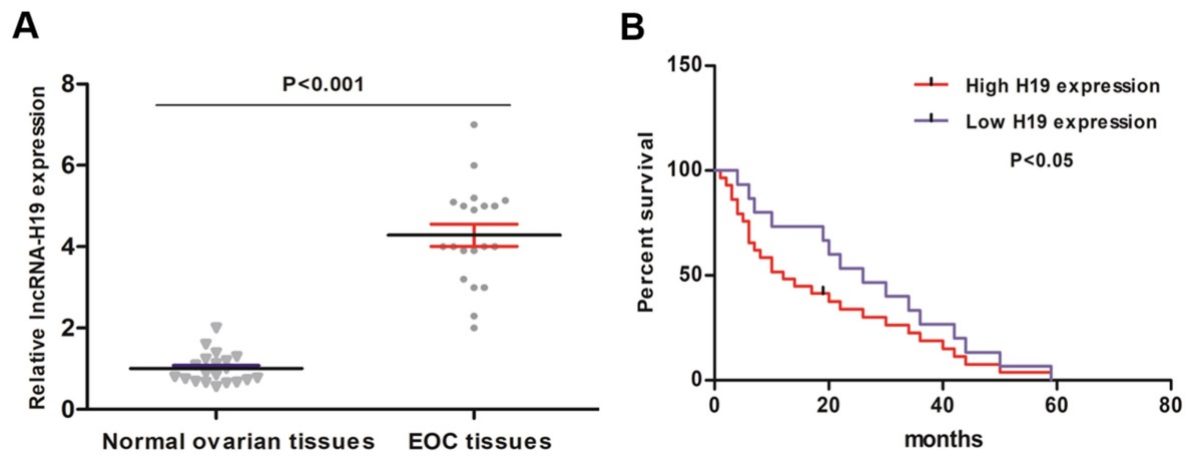
** lncRNA H19 expression characteristics in EOC and its correlation with EOC prognosis.** A) lncRNA-H19 levels were detected by qRT-PCR in 20 primary EOC tissues and normal ovarian tissues. Data are shown as the mean ± SEM. B) lncRNA-H19 was considered high when the expression of lncRNA-H19 was greater than the average, and lncRNA-H19 was regarded as low when the expression of lncRNA-H19 was lower than the average. The Kaplan-Meier curve showing the overall survival of EOC patients with high/low lncRNA-H19 levels was established.

20 primary EOC tissues and 20 normal ovarian tissues were collected and analyzed for lncRNA-H19 levels. The qRT-PCR results indicated that the lncRNA-H19 levels were higher in the EOC tissues than those in the normal ovarian tissues (Fig. 1A). We then employed Kaplan-Meier analysis to evaluate the correlation between lncRNA-H19 levels and EOC patient survival. The EOC patients were categorized into high lncRNA-H19 expression group (lncRNA-H19 expression > median) and low lncRNA-H19 expression group (lncRNA-H19 expression ≤ median). EOC patients with high lncRNA-H19 levels showed poor survival (Fig. 1B). These results indicated lncRNA-H19's involvement in EOC development.

### LncRNA-H19 strengthened ovarian cancer cell resistance to carboplatin

**Figure 2:**
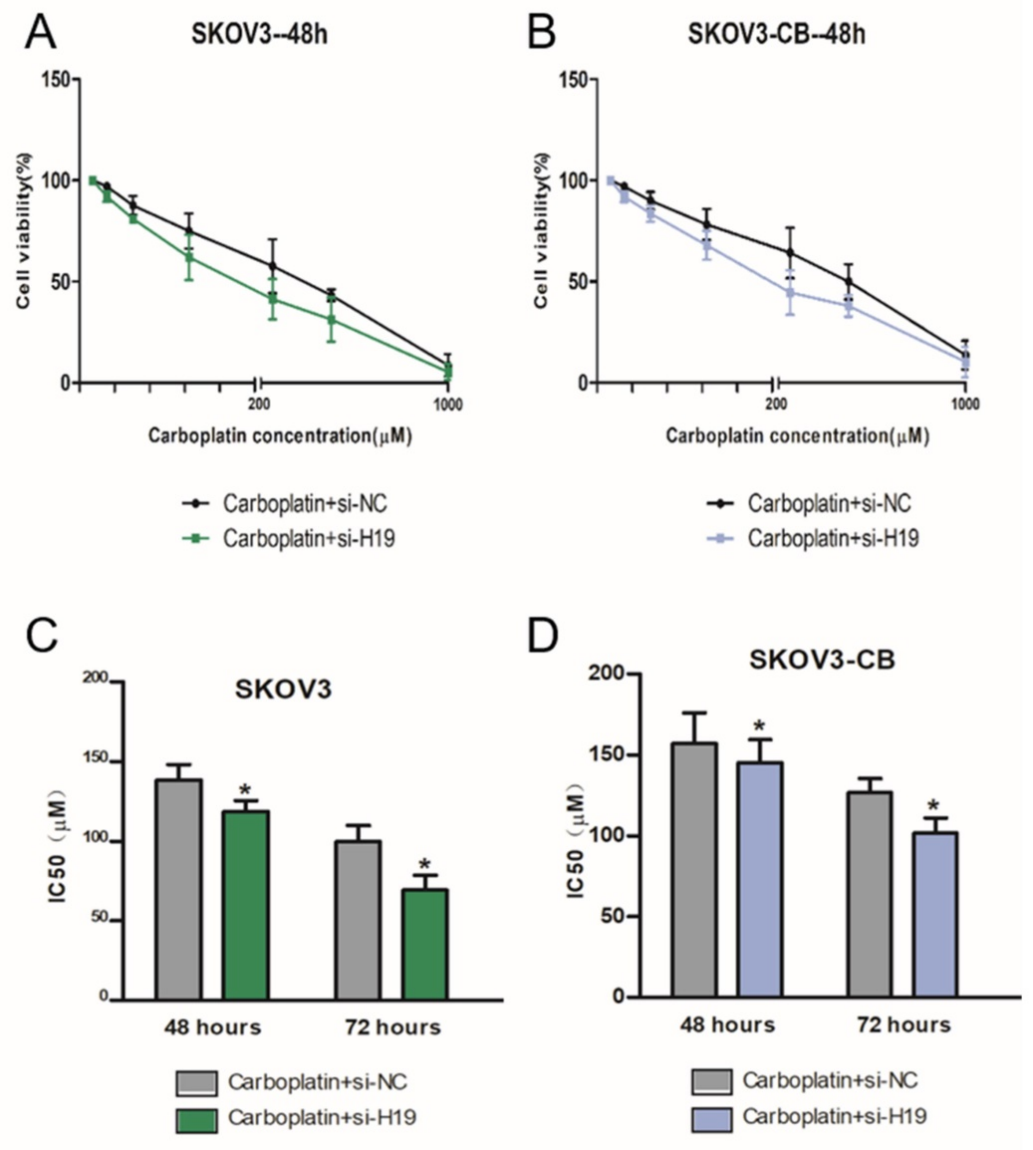
** lncRNA-H19 strengthened ovarian cancer cell resistance to carboplatin.** A-D) SKOV and SKOV3-CB cells were transfected with si-lncRNA-H19 or the negative control and treated with different concentrations of carboplatin for 48 h. Cell viability was measured by CCK-8 assays; E-H) IC50 value for carboplatin in 2 types of EOC cells transfected with si-lncRNA-H19 or negative control and treated with different concentrations determined using CCK-8 assays. Data are shown as the means ± SEM. * p<0.05 versus the negative control.

We then tested the cytotoxic effects of carboplatin on two matched human ovarian cancer cell lines: SKOV3 (wild type), and SKOV3-CB (SKOV3 after long-term carboplatin treatment). Increasing concentrations of carboplatin were given, and cell numbers after 48 h were measured by colorimetric assays. A carboplatin concentration-dependent decrease in viability was observed in both EOC cells (Fig. 2A-B). The relative IC50 of carboplatin was measured at 48 h and 72 h after incubation. As shown in Fig. 2C-D, SKOV3-CB cells had a higher IC50 value than SKOV3 cells. To test whether lncRNA-H19 regulates EOC cell sensitivity to carboplatin, we treated EOC cells with different concentrations of carboplatin with 50 µM of si-lncRNA-H19. Intriguingly, si-lncRNA-H19 substantially lowered the viability of carboplatin-resistant cells incubated with carboplatin with a decrease in the IC50 (Fig. 2C-D). Thus, lncRNA-H19 potently strengthened the chemoresistance of carboplatin-resistant EOC cells. lncRNA-H19 knockdown in response to specific lncRNA-H19 siRNAs is shown in Fig. S1. Two lncRNA-H19 siRNAs were used in preliminary experiments, and siRNA 1 was chosen and used in subsequent experiments.

### LncRNA-H19 and miR‑29b‑3p levels between EOC and control tissues/cells

**Figure 3:**
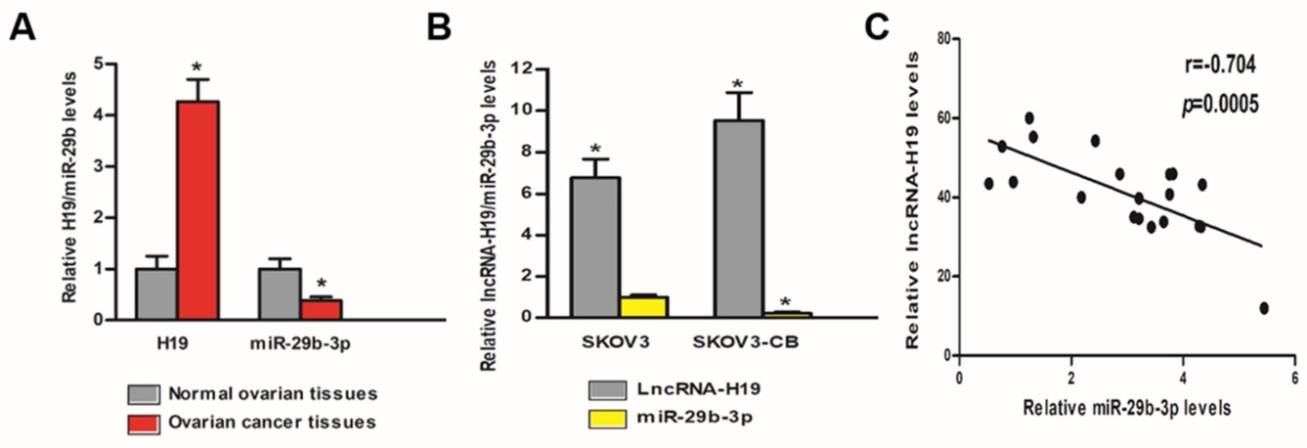
** lncRNA-H19 and miR‑29b‑3p levels between EOC and control tissues/cells.** A) lncRNA-H19 and miR-29b-3p levels were compared between EOC tissues and normal ovarian tissues. * P<0.05 vs. normal ovarian tissues; B) lncRNA-H19 and miR‑29b‑3p expression were compared in SKOV3 and SKOV3-CB cell lines. * lncRNA-H19 level P<0.05 vs. SKOV3 cell line, ^#^ miR-29b-3p level P<0.05 vs. SKOV3 cell line; C) lncRNA-H19 and miR-29b-3p levels in EOC tissues were negatively correlated.

We tested the lncRNA-H19 and miR-29b-3p levels in the tissues and cells of EOC patients and healthy controls. Table 1 shows the pathological features of the EOC patients included in the study. As shown in Fig 3A, lncRNA-H19 expression was much higher in the EOC tissues than that in the normal ovarian tissues (P<0.05), and miR‑29b‑3p expression in the EOC tissues was much lower than that in the normal ovarian tissues (P<0.05). Furthermore, lncRNA-H19 expression in SKOV3-CB cells was higher than that in SKOV3 cells (P<0.05); however, miR‑29b‑3p was significantly lower in SKOV3-CB cells was higher than that in SKOV3 cells (P<0.05) (Fig. 3B). To explore the miR-29b-3p/lncRNA-H19 correlation, we measured the expression levels of miR-29b-3p and lncRNA-H19 in the sera of 20 patients. A negative correlation was found between the two ncRNAs (r=-0.704; Fig. 3C).

### LncRNA-H19 sponged miR‑29b‑3p in EOC cells

**Figure 4:**
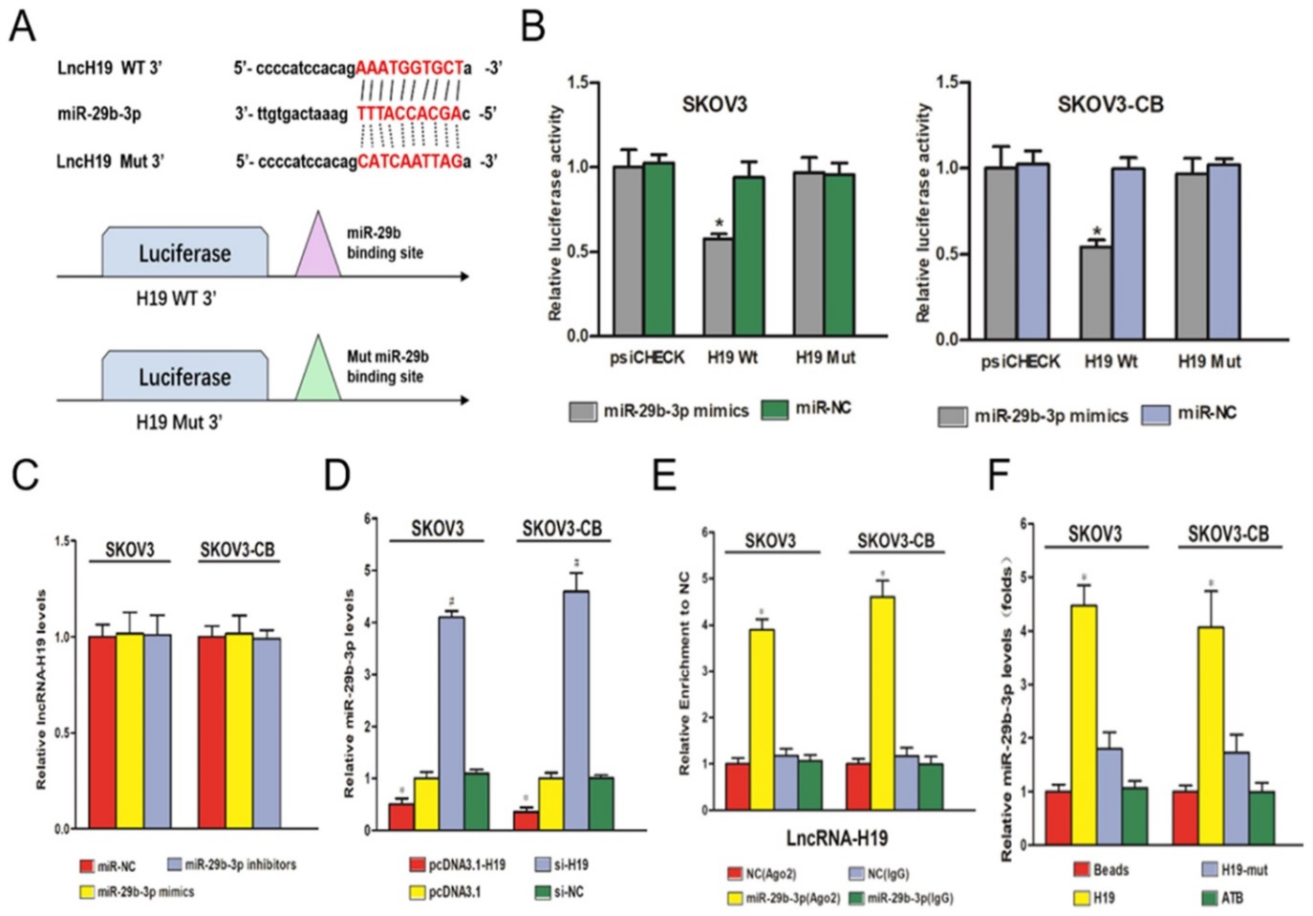
** lncRNA-H19 sponged miR‑29b‑3p in EOC cells.** A) lncRNA-H19 is targeted by miR‑29b‑3p; B-C) Luciferase activities in the SKOV3 and SKOV3-CB cell lines were compared between different transfection groups. * P<0.05 vs. the miR-29b-3p mimic+H19-Mut group; D) Influence of miR‑29b‑3p on lncRNA-H19 expression was determined in the SKOV3 and SKOV3-CB cell lines; E) Influence of lncRNA-H19 on miR‑29b‑3p expression was determined in the SKOV3 and SKOV3-CB cell lines; F) Ago2-RIP followed by miRNA RT-qPCR to detect the endogenous miR-29b-3p association with the Ago2-tagged lncRNA-H19 transcript; G) RNA pull-down assays were performed in EOC cells. lncRNA-H19 levels were measured by RT-qPCR. * P<0.05 vs. the negative control. miR/miRNA, microRNA; si, short interfering RNA; NC, negative control; ncRNA, noncoding RNA; lncRNA, long noncoding RNA; RIP, RNA binding protein immunoprecipitation.

A major mechanism of lncRNAs is to competitively bind to its target miRNAs, which buffers miRNAs' inhibitory effect on their downstream targets. The negative correlation between lncRNA-H19 and miR-29b-3p suggested a potential negative regulatory mechanism between the two ncRNAs. Starbase 2.0 was employed to predict targeting sites for lncRNA-H19 and miR-29b-3p (Fig. 4A). A dual‑luciferase reporter gene assay showed that compared with the cells transfected with pmirGLO-H19-Wt and miR-NC, the cells transfected with pmirGLO-H19-Wt and miR-29b-3p mimics showed a strong decrease in luciferase activity (P<0.05) (Fig. 4B). No significant change in luciferase activity was found in the pmirGLO-H19-Mut+miR-29b-3p mimic group and the pmirGLO-H19-Wt+miR-NC group (Fig. 4B). Furthermore, RT‑qPCR experiments showed that a change in the miR-29b-3p level did not change lncRNA-H19 expression (Fig. 4C). However, higher lncRNA-H19 levels dramatically decreased miR-29b-3p levels in EOC cell lines (P<0.05) (Fig. 4D). These results indicated that lncRNA-H19 is an upstream regulator of miR-29b-3p. Ago2-RIP was used to validate the direct interaction of lncRNA-H19 and miR-29b-3p, which indicated that the Ago2-tagged wild-type lncRNA-H19 was observably enriched for miR-29b-3p compared to the empty vector and lncRNA-H19 with a mutated binding site (Fig. 4E). Further, the results of the RNA pulldown assay showed that lncRNA-H19 was pulled down by biotin-labeled miR-29b-3p oligos (Fig. 4F). Together, these results indicated that lncRNA-H19 served as a sponging lncRNA for miR-29b-3p.

### LncRNA H19 attenuated miR-29b-3p-mediated carboplatin sensitivity in carboplatin-resistant EOC cells

**Figure 5:**
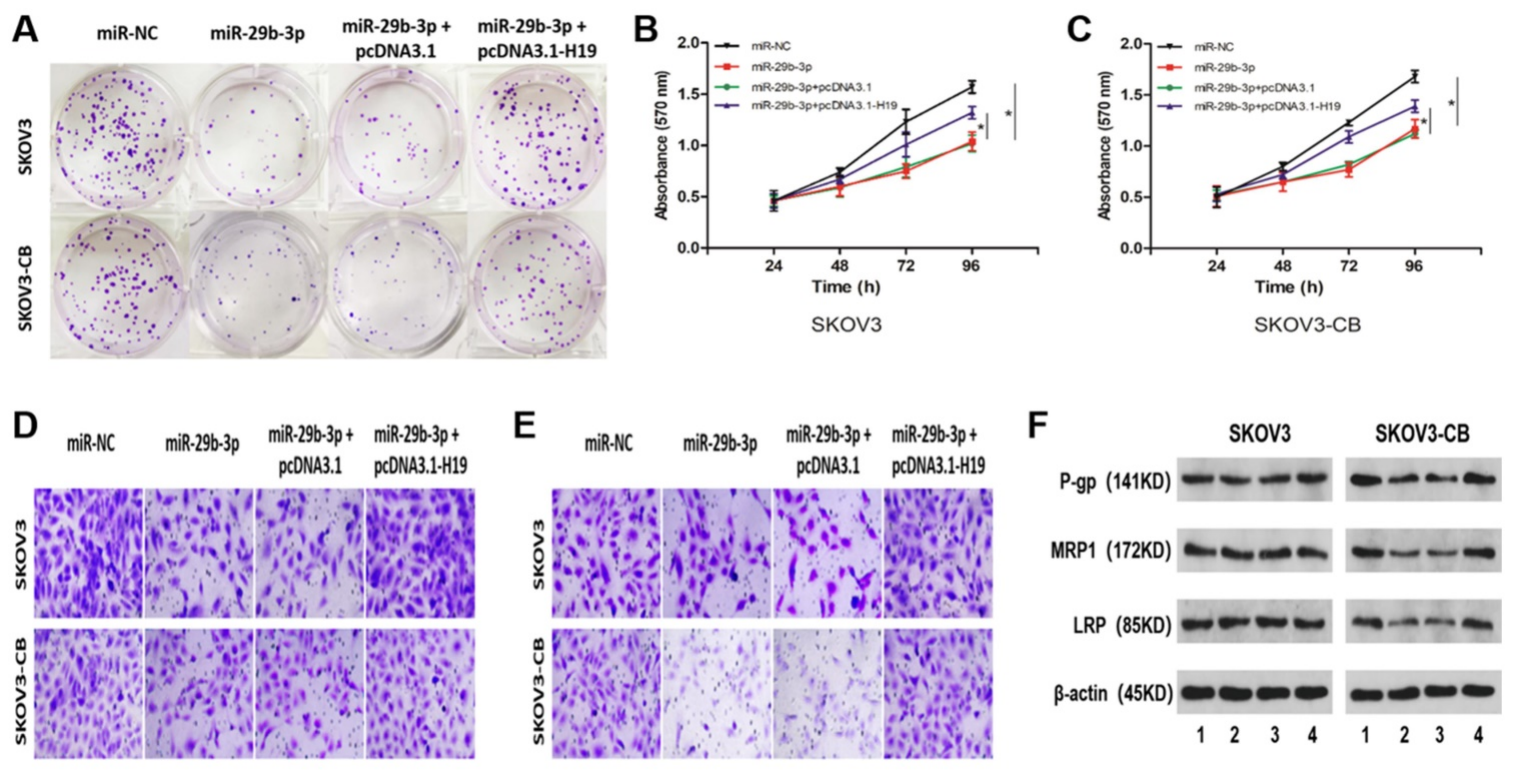
** lncRNA-H19 attenuated miR-29b-3p-mediated carboplatin sensitivity in EOC cells.** SKOV3 and SKOV3-CB cells were transfected with miR-NC, miR-29b-3p, miR-29b-3p+pcDNA3.1, or miR-29b-3p+pcDNA3.1-H19, followed by detection of colony number (A, 200×), cell proliferation (B and C), migration (D, 200×), invasion (E, 200×), and the drug-resistance-related proteins P-gp, MRP1, and LRP (F and G). *P<0.05. IC50, 50% inhibitory concentration. For Figure 5F, lane 1-4 stands for miR-NC, miR-29b-3p, miR-29b-3p + pcDNA3.1, and miR-29b-3p + pcDNA3.1-H19, respectively.

To further determine whether the lncRNA-H19/miR-29b-3p axis affects chemosensitivity in EOC cells, we carried out rescue experiments by transfecting miR-NC, miR-29b-3p, miR-29b-3p+pcDNA3.1, or miR29b-3p+pcDNA3.1-H19 into SKOV3/SKOV3-CB cells. Overexpression of miR-29b-3p inhibited colony formation (Fig. 5A), cell proliferation (Fig. 5B and 5C), migration (Fig. 5D), and invasion (Fig. 5E). Interestingly, no significant difference was seen in the abovementioned cellular phenotypes between the two cell lines. However, western blot results indicated that exogenous expression of miR-29b-3p downregulated the drug resistance-associated proteins P-gp, MRP1, and LRP (Fig. 5F) in carboplatin-resistant SKOV3-CB cells. Moreover, restoration of H19 blocked all of the three miR-29b-3p-manipulated protein levels in carboplatin-resistant EOC cells (Fig. 5F). Collectively, these findings showed that lncRNA-H19 attenuated reactivity to carboplatin by blocking miR-29b-3p in carboplatin-resistant EOC cells.

### STAT3 was a direct target of miR-29b-3p and was negatively regulated by miR-29b-3p in EOC cell lines

**Figure 6:**
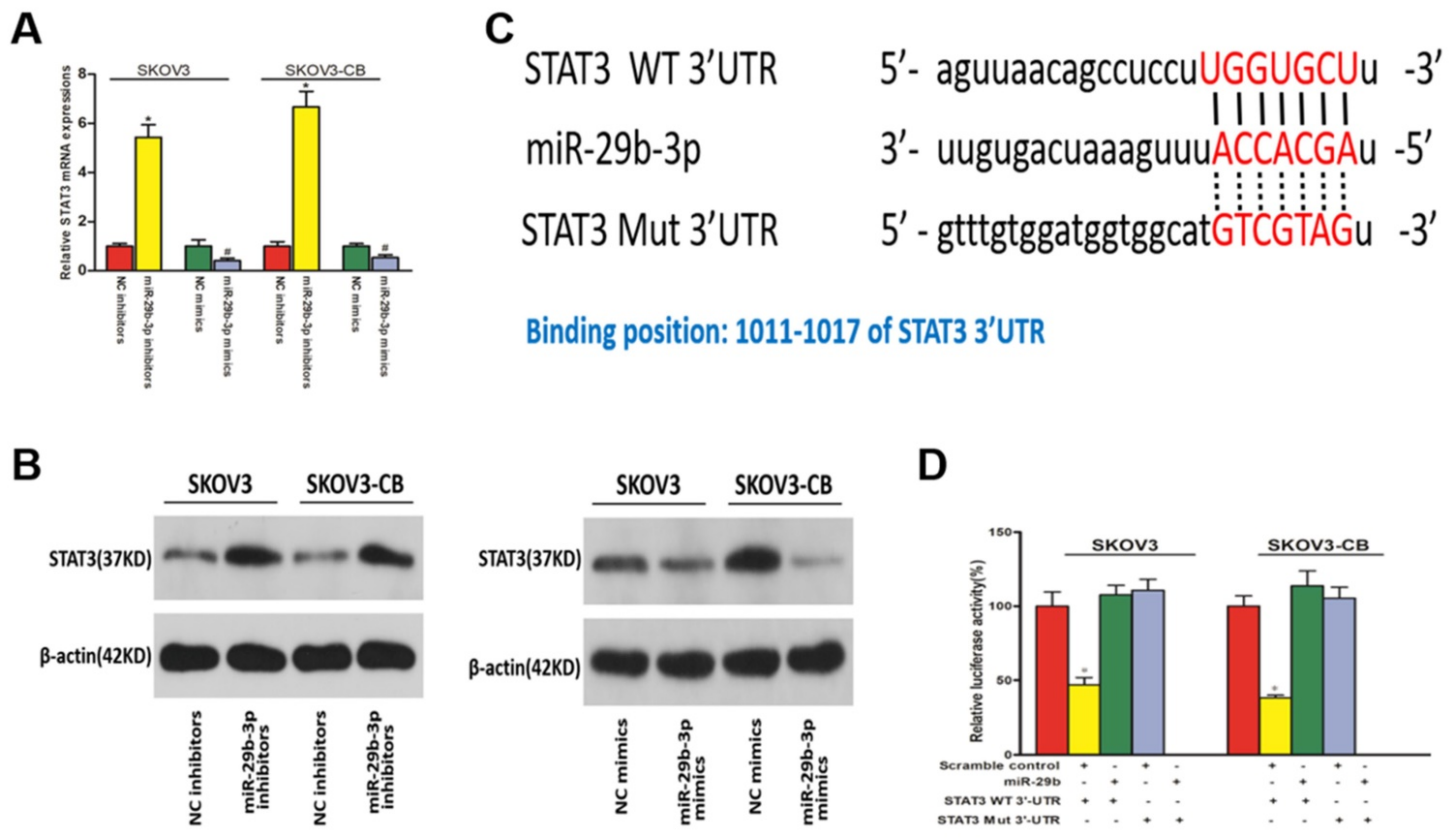
** STAT3 was downregulated in EOC cells and was a downstream target of miR-29b-3p.** qPCR (A) and Western blot (B) results show that miR-29b-3p mimics and inhibitors lowered and increased STAT3 expression, respectively, at both the mRNA and protein levels. C) Schematic of the STAT3 3'UTR luciferase reporter system. D) Luciferase reporter assay results confirmed the direct binding between miR-29b-3p and the STAT3 3'UTR. Data are presented as the mean ± SEM. * P<0.05 versus the control.

We used miRanda and TargetScan software to predict the downstream targets of miR-29b-3p associated with EOC chemoresistance. STAT3 was identified as a potential target of miR-29b-3p considering its role in chemoresistance. As shown in Fig. 6A and 6B, miR-29b-3p mimic and inhibitor transfection caused decreased and increased STAT3 expression, respectively, at both the mRNA and protein levels. Therefore, STAT3 was negatively regulated by miR-29b-3p in EOC cell lines. miR-29b-3p showed complementarity with the STAT3 3'UTR sequence (Fig. 6C). This observation suggested that STAT3 might be inhibited by miR-29b-3p through complementary binding to its 3'UTR sequences. Finally, a 3'UTR luciferase reporter assay confirmed the direct binding between miR-29b-3p and the STAT3 3'UTR. Decreased luciferase expression was only observed in the cells transfected with the wild-type STAT3 3'UTR, and this suppressive effect of miR-29b-3p was blocked by miR-29b-3p binding site mutations in the STAT3 3'UTR (Fig. 6D). Collectively, these data suggested that miR-29b-3p negatively regulated STAT3 expression by complementary binding.

### The lncRNA H19-miR-29b-3p-STAT3 axis regulated carboplatin sensitivity in EOC cells

**Figure 7:**
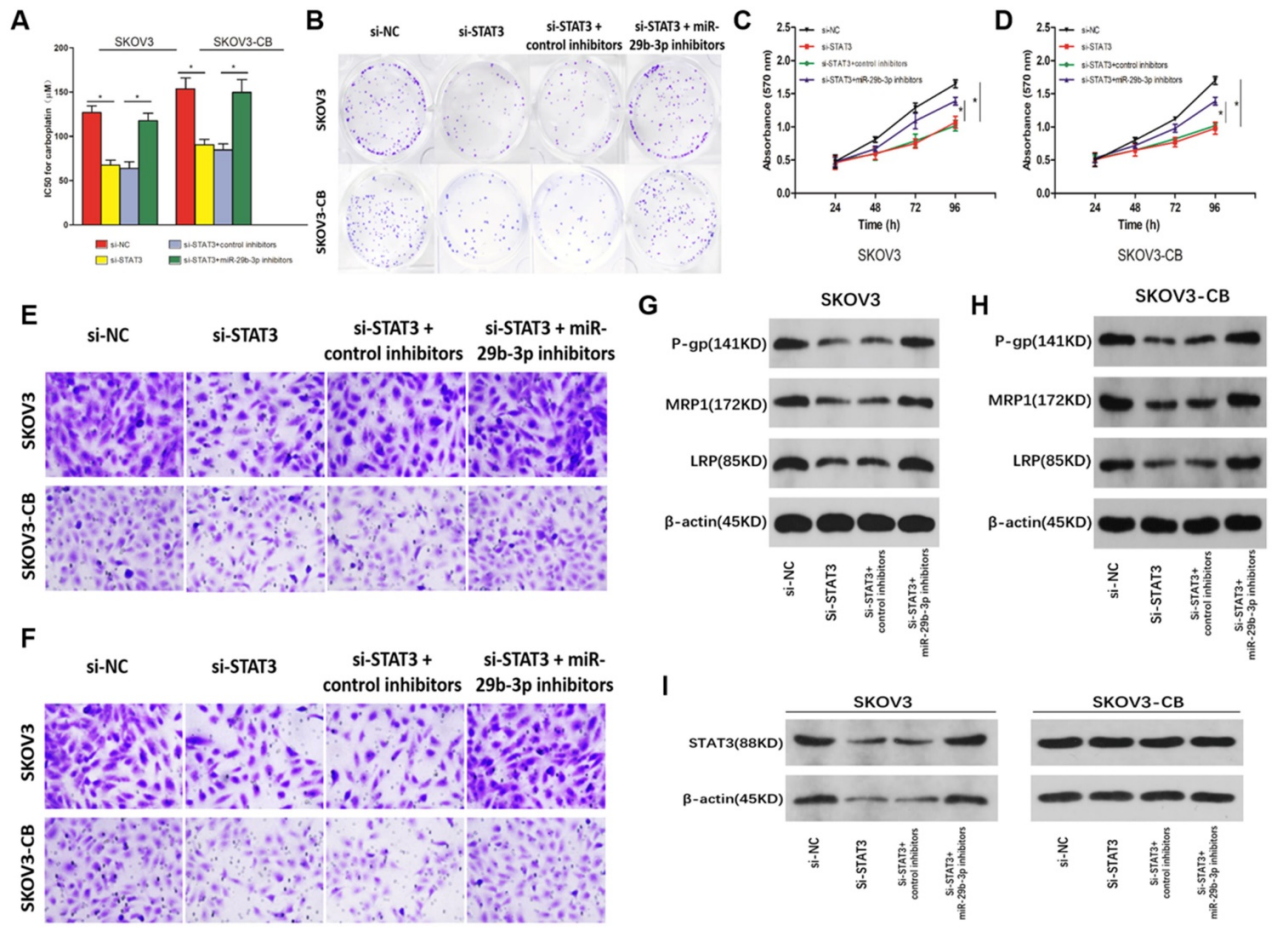
** Role of the lncRNA-H19-miR-29b-3p-STAT3 axis in the carboplatin sensitivity of EOC cells.**EOC cells were transfected with si-NC, si-STAT3, si-STAT3+anti-miR-NC, or si-STAT3+anti-miR-29b-3p, and then, the IC50 value for carboplatin was measured in each group (A), along with colony number (B, 200×), cell proliferation (C and D), migration (E, 200×), invasion (F, 200×), and P-gp, MRP1, and LRP proteins (G and H); I) STAT3 expression in SKOV3 and SKOV3-CB cells following the transfection of si-NC, si-STAT3, si-STAT3+control inhibitors, or si-STAT3+miR-29b-3p inhibitors. *P<0.05.

Rescue experiments were carried out in SKOV3/SKOV3-CB cells to explore the correlation between miR-29b-3p and STAT3 in the drug sensitivity of EOC cells. STAT3 knockdown increased the sensitivity of EOC cells to carboplatin, as indicated by the lower IC50 values (Fig. 7A) and colony formation (Fig. 7B), inhibited cell proliferation (Fig. 7C and 7D), migration (Fig. 7E), and invasion (Fig. 7F), and lower levels of drug-resistance-associated proteins P-gp, MRP1, and LRP (Fig. 7G and 7H) in both cell lines. However, inhibition of miR-29b-3p induced si-STAT3-mediated drug sensitivity in EOC cells (Fig. 5). To determine whether lncRNA-H19 regulated carboplatin resistance via the miR-29b-3p/STAT3 axis, we transfected si- lncRNA-H19 into EOC cells together with control inhibitors or miR-29b-3p inhibitors. As shown in Fig. 7I, knockout of lncRNA-H19 significantly decreased STAT3 expression, which was restored by miR-29b-3p inhibition. Collectively, our findings indicated that the lncRNA-H19/miR-29b-3p/STAT3 axis is a regulatory pathway in the carboplatin resistance of EOC cells.

## Discussion

Carboplatin is a widely used chemotherapeutic drug for EOC, but the development of carboplatin resistance substantially limits the treatment effect of chemotherapy. A growing number of studies have proven that lncRNA dysregulation is a possible reason for chemoresistance in different human cancers, including EOC 15 16. Identification of novel EOC-specific chemoresistance-associated lncRNAs is important for the future identification of lncRNA-targeted treatment. Here, we found that lncRNA-H19 was highly expressed in carboplatin-resistant EOC tumor tissues and cells. Admittedly, the sample size and control size in the current study are relatively small, and future expansion of sample size is in need to illustrate the study strength. Further, we proposed for the first time that lncRNA-H19 knockdown improved the sensitivity of EOC cells to carboplatin by suppressing STAT3 by functioning as a ceRNA of miR-29b-3p.

Currently, lncRNA-H19 was observed to regulate cell survival, migration, invasion and apoptosis by regulating target microRNAs 17 18. Various functional mechanisms have been identified between lncRNAs and miRNAs in tumor biology. The most studied functional mechanism for lncRNAs is acting as ceRNAs to block the binding of specific miRNAs and their target genes, which contributes to target mRNA derepression. In our study, we aimed to explore the functional mechanism of lncRNA-H19 in the carboplatin resistance of EOC. As a result, miR-29b-3p was observed to be a lncRNA-H19 target. Rescue experiments further demonstrated that miR-29b-3p sensitized EOC cells to carboplatin, and the effect of miR-29b-3p was reversed by lncRNA-H19 overexpression. Considered a versatile player in cancer development and progression, miR-29b-3p has been found to play a central role in the cancer response to chemotherapy in different cancer types 19 20 21. However, no study has shown whether miR-29b-3p participates in the regulation of chemotherapy in EOC. Our study is the first to reveal the function and underlying mechanism of miR-29b-3p in enhancing carboplatin sensitivity in carboplatin-resistant EOC cells.

miRNAs are reported to negatively regulate target genes through binding with their 3ʹUTRs. In the current study, we showed that miR-29b-3p negatively regulates signal transducer and activator of transcription 3 (STAT3) by directly targeting their 3'UTR sequences, enriching our understanding of miR-29b-3p as a chemoresistance regulator in EOC development. STAT3 is a well-known oncogenic transcription factor and is increasingly associated with cancer initiation, progression, metastasis, chemoresistance, and immune evasion 22 23. STAT3 signaling has been found to negatively regulate the anticancer activity of chemotherapies in different cancers, including EOC 24 25 26. Here, we found that knockdown of STAT3 enhanced the chemosensitivity of EOC cells to carboplatin, and the biological function of STAT3 was rescued by miR-29b-3p inhibitors. Moreover, lncRNA-H19 is a miR-29b-3p sponge, and lncRNA-H19 silencing repressed the miR-29b-3p downstream target STAT3. In addition, a recently published study observed that lncRNA-H19/miR-29b-3p/STAT3 signaling contributed to lung cancer progression by mediating cell viability, survival, apoptosis and the EMT process 14. Our and other findings indicated that the specific role and underlying molecular mechanism of the lncRNA-H19/miR-29b-3p/STAT3 pathway in the initiation and development of cancer should be further explored in the future.

## Conclusion

This study revealed that lncRNA-H19 and STAT3 were significantly increased but miR-29b-3p was decreased in carboplatin-resistant EOC. Silencing lncRNA-H19 improved carboplatin effectiveness in EOC cells. LncRNA-H19 is a competing endogenous RNA of miR-29b-3p that further regulates the miR-29b-3p downstream target STAT3, and STAT3 eventually leads to resistance to carboplatin in EOC cells. In conclusion, we propose that the lncRNA-H19/miR-29b-3p axis enhances carboplatin resistance in EOC by targeting STAT3 and may provide a possible way to improve chemotherapy for EOC.

## Supplementary Material

Supplementary figure.

## Figures and Tables

**Table 1:** Correlation between H19 expression and clinicopathologic characteristics of EOC patients.

Clinicopathologic Parameters	H19 expression		*P*
	High	Low	
Age (years)			
55 and above	8	6	0.3291
Under 55	2	4	
FIGO stage			
I-II	3	8	0.0246
III-IV	7	2	
Differentiation			
Well and moderate	7	1	0.0062
Poorly	3	9	
Lymph node metastasis			
Absent	4	7	0.1775
Present	6	3	

FIGO, International Federation of Gynecology and Obstetrics stage.
