# Supplementary figures and images for: LncRNA-H19 regulates chemoresistance to carboplatin in epithelial ovarian cancer through microRNA-29b-3p and STAT3
Source: J Cancer. 2021 Jul 25;12(19):5712–22. doi: 10.7150/jca.58979 (PMC8408112; doi:10.7150/jca.58979)

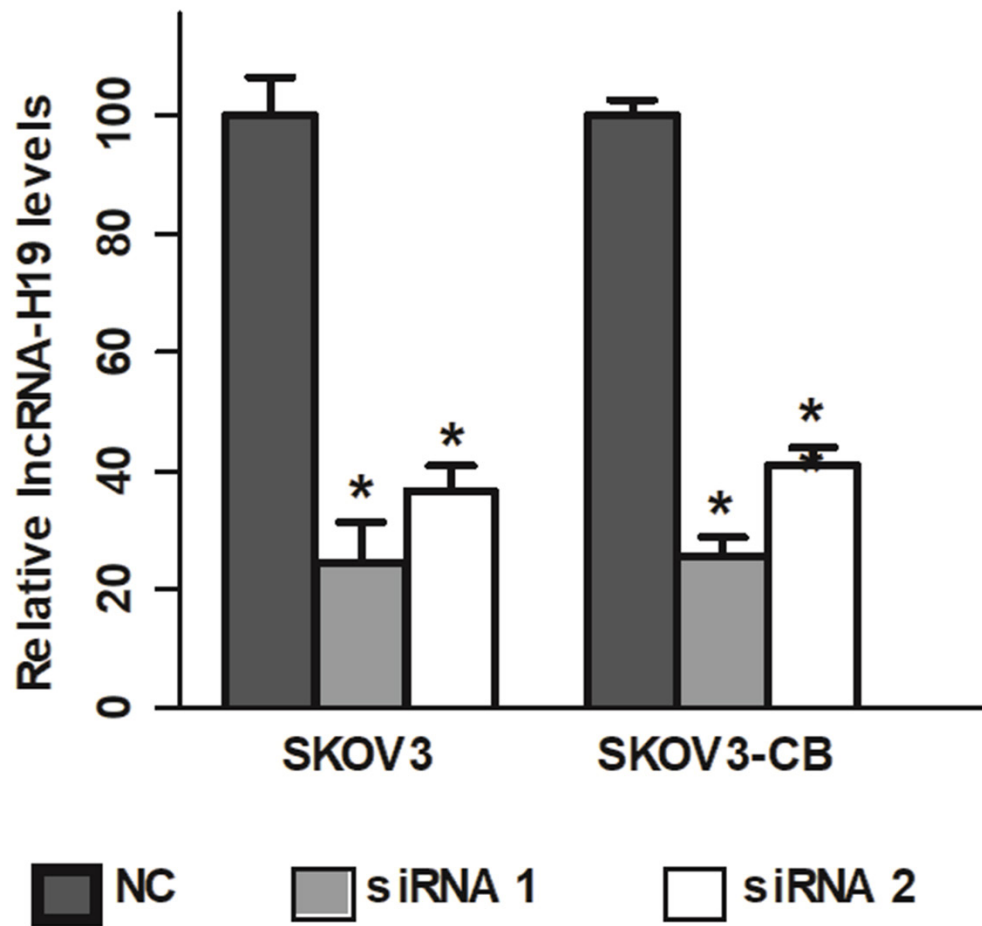

Figure S1: knockdown efficiency of lncRNA-H19 by si-H19.

Supplement: Supplementary file 1 — Supplementary figure. [file jcav12p5712s1.pdf]
